# Induction of apoptosis in human prostate cancer cell line, PC3, by 3,3′-diindolylmethane through the mitochondrial pathway

**DOI:** 10.1038/sj.bjc.6602145

**Published:** 2004-08-24

**Authors:** M Nachshon-Kedmi, S Yannai, F A Fares

**Affiliations:** 1 Faculty of Food Engineering and Biotechnology, Technion-Israel Institute of Technology, Haifa 32000, Israel; 2 Department of Biochemistry and Molecular Genetics, Carmel Medical Center, Haifa, Israel; 3 Faculty of Medicine, Technion-Israel Institute of Technology, Haifa, Israel

**Keywords:** 3,3′-diindolylmethane, apoptosis, caspases, PC3 cells, prostate cancer

## Abstract

Prostate cancer is the most common malignancy and the second leading cause of male death in Western countries. Prostate cancer mortality results from metastases to the bones and lymph nodes and progression from androgen-dependent to androgen-independent disease. Although androgen ablation was found to be effective in treating androgen-dependent prostate cancer, no effective life-prolonging therapy is available for androgen-independent cancer. Epidemiological studies have shown a strong correlation between consumption of cruciferous vegetables and a lower risk of prostate cancer. These vegetables contain glucosinolates, which during metabolism give rise to several breakdown products, mainly indole-3-carbinol (I3C), which may be condensed to polymeric products, especially 3,3′-diindolylmethane (DIM). It was previously shown that these indole derivatives have significant inhibitory effects in several human cancer cell lines, which are exerted through induction of apoptosis. We have previously reported that I3C and DIM induce apoptosis in prostate cancer cell lines through p53-, bax-, bcl-2- and fasL-independent pathways. The objective of this study was examination of the apoptotic pathways that may be involved in the effect of DIM in the androgen-independent prostate cancer cell line, PC3, *in vitro*. Our results suggest that DIM induces apoptosis in PC3 cells, through the mitochondrial pathway, which involves the translocation of cytochrome *c* from the mitochondria to the cytosol and the activation of initiator caspase, 9, and effector caspases, 3 and 6, leading to poly ADP-ribose polymerase (PARP) cleavage and induction of apoptosis. Our findings may lead to the development of new therapeutic strategies for the treatment of androgen-independent prostate cancer.

Prostate cancer is the most common diagnosed malignancy and the second leading cause of male death in Western countries (Tang and Porter, 1997; Crawford, 2003). Mortality from prostate cancer results from metastases to the bones and lymph nodes and progression from androgen-dependent disease to androgen-independent prostatic growth (Bruckheimer and Kyprianou, 2000). The androgen-dependent phase of prostate cancer may be effectively treated with androgen ablation therapies, which cause involution of the prostate gland, as a result of inhibition of cellular proliferation and stimulation of apoptosis (Tang and Porter, 1997). However, in most of the patients, progression to the lethal stage of androgen independence, for which there is no effective life-prolonging therapy, eventually occurs (Tang and Porter, 1997; Abate-Shen and Shen, 2000). Hence, intense investigations emerge, trying to better understand androgen-independent disease and seeking effective means against this type of cancer.

Several epidemiological studies have shown a strong correlation between consumption of diets rich in fruits and vegetables and a lower risk of various cancers (Dragsted *et al*, 1993; Tavani and La Vecchia, 1995). A number of studies have demonstrated a reduced risk of developing prostate cancer in humans consuming cruciferous vegetables, such as broccoli, Brussels sprouts, cabbage and cauliflower (Jain *et al*, 1999; Cohen *et al*, 2000; Kolonel *et al*, 2000). These vegetables contain glucosinolates which, during metabolism, give rise to several breakdown products, mainly indole-3-carbinol (I3C) (Bradfield and Bjeldanes, 1987; Verhoeven *et al*, 1997). In a low pH environment, I3C is converted into polymeric products, among which 3,3′-diindolylmethane (DIM) is the main one (Bradfield and Bjeldanes, 1987; De Kruif *et al*, 1991). It was previously shown that these indole derivatives have inhibitory effects on the viability and proliferation of several human cancer cell lines (Ge *et al*, 1996; Fares *et al*, 1998; Bonnesen *et al*, 2001; Chen *et al*, 2001; Chinni *et al*, 2001; Kedmi *et al*, 2003). These studies demonstrated that the indolic compounds exert their effects in the cancer cells through the induction of an apoptotic cell response.

Apoptosis, a programmed cell death, is critical for normal development, function and homeostasis of multicellular organisms, and is regulated by the expression of multiple genes such as p53, bcl-2 family members and cytochrome *c* (Cosulich *et al*, 1999; Sheikh and Fornace, 2000). Signalling for apoptosis occurs through multiple independent pathways that are initiated by diverse extracellular and intracellular factors (Strasser *et al*, 2000). Activation of a family of cysteine proteases, caspases, plays a critical role in the execution of all apoptosis signalling pathways (Nunez *et al*, 1998; Strasser *et al*, 2000). These enzymes cleave vital cellular proteins, resulting in distinct biochemical and morphological features, including cell shrinkage, chromatin condensation and DNA fragmentation (Bortner *et al*, 1995; Strasser *et al*, 2000).

We and others (Chinni *et al*, 2001; Kedmi *et al*, 2003) have previously shown that indole derivatives originating from cruciferous vegetables, I3C and DIM, have significant inhibitory effects in prostate cancer cell lines, which are carried out by induction of apoptosis. The purpose of our current study was to examine the apoptotic pathways that may be involved in the effect of DIM in the androgen-independent prostate cancer cell line, PC3, *in vitro*.

## MATERIALS AND METHODS

### Materials

3,3′-Diindolylmethane was purchased from Designed Nutritional Products (USA). Cell culture media and reagents were obtained from Biological Industries (Beit Haemek, Israel). Anti-caspase 3 and anti-cytochrome *c* monoclonal antibodies were purchased from Santa Cruz Biotechnology (Santa Cruz, CA, USA). Anti-caspase 6 and 9 monoclonal antibodies were purchased from Medical and Biological Laboratories (Japan). Anti-caspase 8 monoclonal antibody was purchased from Oncogene (Boston, MA, USA). Anti-actin monoclonal antibody was purchased from ICN Biomedicals (Aurora, OH, USA). Secondary antibody peroxidase-conjugated goat anti-mouse IgG was purchased from Jackson Immune Research Laboratories (West Grove, PA, USA). Colorimetric kits for the detection of caspase activity were purchased from Calbiochem (San Diego, CA, USA). All other chemicals were purchased from Sigma or other local sources.

### Cell culture

Human prostate cancer cell line, PC3 (deficient in p53 gene, androgen-independent, poorly differentiated), was obtained from the American Type Culture Collection, Manassas, VA, USA. Cells were grown in F-12 medium supplemented with 10% heat-inactivated foetal calf serum, and 100 U ml^−1^ penicillin/streptomycin (Beit Haemek, Israel). Cells were cultured at 37°C in an atmosphere of 95% air and 5% of CO_2_.

### 3,3′-Diindolylmethane stock solution

3,3′-Diindolylmethane stock solution was prepared by dissolving DIM powder in DMSO to yield a final concentration of 0.1 M. The final concentration of DMSO in the culture medium was 0.08% (v v^−1^). This concentration of DMSO was established as nontoxic to any cell line.

### Total protein extraction

PC3 cells were treated with 10 ml of culture medium containing 75 *μ*M DIM for 8, 16, 24, 48 and 72 h. The control cells were treated with 0.08% (v v^−1^) of DMSO solution. At the end of each treatment, cells were collected and their total protein fraction was extracted as described previously (Kedmi *et al*, 2003). Protein concentrations of the cell lysates were quantified by the method of Bradford (1976).

### Cytosol protein extraction

PC3 cells were treated with 75 *μ*M DIM for 8, 16, 24, 48 and 72 h. At the end of each treatment, cells were harvested and their cytosol proteins were extracted, as described earlier (Bossy-Wetzel *et al*, 1998). Total cytosol proteins were precipitated with 10% TCA and harvested by centrifugation at 10 000 **g** and 4°C for 15 min. The pellets were resuspended in 0.4 M NaOH to give a final protein concentration of 3 mg ml^−1^, as described previously (Grubb *et al*, 2001).

### Western blot analysis

In all, 60 *μ*g of protein from each sample were separated by 15% SDS–polyacrylamide gels (SDS–PAGE), electrophoretically transferred onto a nitrocellulose membrane filter and incubated with specific antibodies, as described earlier (Kedmi *et al*, 2003). Results were quantified by densitometer analysis (Zilber Lurma, France) using Bio1D software, and are expressed as percentages of the respective controls. Actin level (as standard protein occurring naturally in these cells) was used as a reference.

### Caspase colorimetric assay

PC3 cells were treated with 75 *μ*M DIM for 8, 16, 24, 48 and 72 h. At the end of each treatment, cells were collected, proteins were extracted and the activity of caspases 3, 6, 8 and 9 was determined using colorimetric kits, according to the manufacturer's instructions (Calbiochem).

### Statistics

Western blot analyses were repeated three times, and the quantitative evaluation of the protein levels using densitometeric analysis is presented as means±standard error (s.e.). Caspase colorimetric activity determination was repeated three times, each performed in duplicate, and the data are presented as means±s.e. Statistical analyses of the differences between controls and treated groups were performed using Student's *t*-test.

## RESULTS

### Effect of DIM on caspase protein levels and activities

In this study, we examined the effect of DIM on the levels and activities of initiator and effector caspases, in order to better characterise the pathway through which DIM exerts its apoptotic effects in these cells. PC3 cells were treated with 75 *μ*M DIM for 8, 16, 24, 48 and 72 h. At the end of each treatment, cells were harvested and their total protein fraction was extracted. Determination of caspase 3, 6, 8 and 9 levels was conducted using Western blotting analysis with a quantitative analysis of three independent blots, using a densitometer, as described in ‘Materials and methods’.

**Figure 1:**
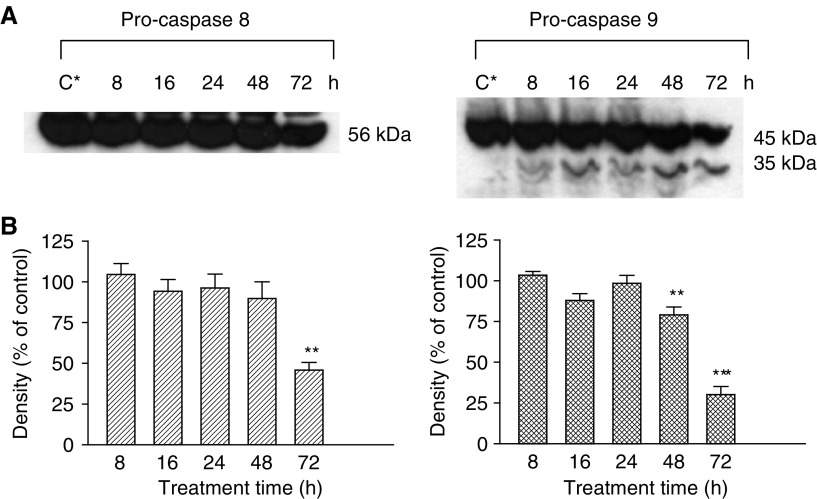
Pro-caspase 8 and 9 levels following treatment of PC3 cells with 75 *μ*M DIM for 8–72 h. Cells were treated with DIM and their total protein fraction was extracted, separated on SDS–PAGE and exposed to specific antibodies using Western blotting, as described in ‘Materials and methods’. (**A**) Western blotting results. C^*^ denotes a representative control group. The figures shown are representatives of three independent experiments. (**B**) Densitometer results. Data presented are averages of three independent experiments (±s.e.) and are expressed as percentages of the respective controls. ^**^P<0.01; ^***^P<0.001.

The Western blot results shown in Figure 1

indicate the levels of the initiator caspases, pro-caspase 8 and 9. Treatment of PC3 cells with DIM for 72 h causes a decrease in pro-caspase 8 (56 kDa) levels (Figure 1A), which was significant, according to the densitometer analysis (*P*<0.01), in comparison with the control (Figure 1B). There was also a significant decrease in pro-caspase 9 (45 kDa) levels in these cells after exposure to DIM for 48 (*P*<0.01) and 72 h (*P*<0.001). Furthermore, the results indicate (Figure 1A) the appearance of one of the active subunits of caspase 9 (35 kDa), when cells were treated with DIM for 8 h. The levels of this subunit increase when the cells are exposed to DIM, in a time-dependent manner. This protein is absent from the control group.

Figure 2A

**Figure 2:**
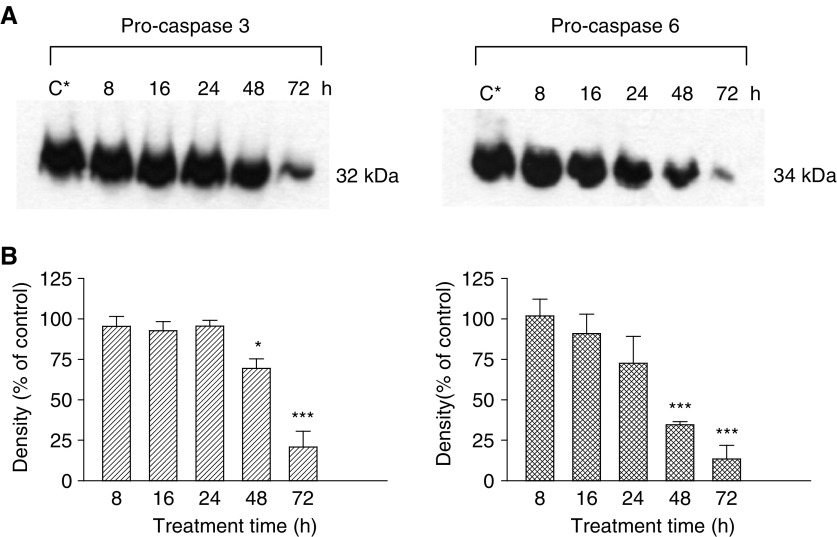
Pro-caspase 3 and 6 levels following treatment of PC3 cells with 75 *μ*M DIM for 8–72 h. The experimental details and the designations of letters (**A**–**C**) were as described for Figure 1. ^*^P<0.05; ^***^P<0.001.

indicates a decrease in the levels of both effector caspases, pro-caspase 3 (32 kDa) and 6 (34 kDa), after exposure to DIM, in a time-dependent manner. Quantitative analysis of the results observed using a densitometer revealed (Figure 2B) that this decrease was significant when cells were exposed to DIM for 48 or 72 h (*P*<0.001) in comparison with the controls.

**Figure 3:**
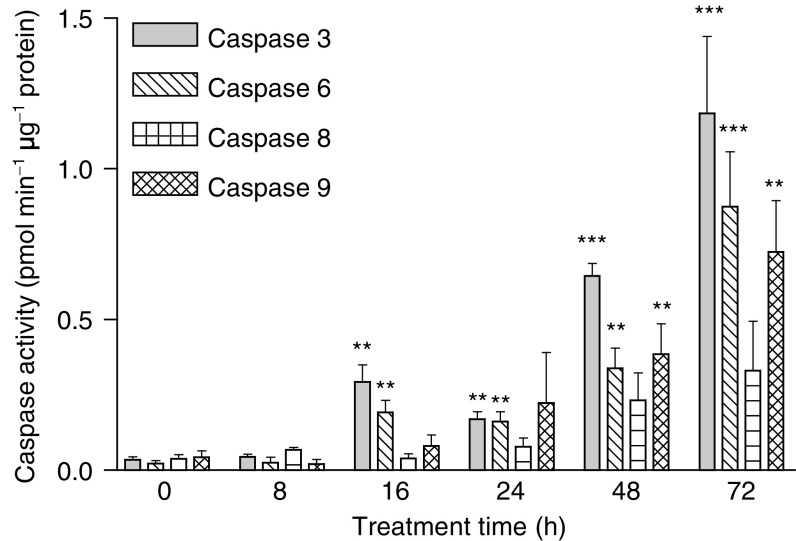
Effect of DIM on caspase activation in PC3 cells. Determination of enzyme activity was applied by a colorimetric assay using specific caspase substrates, as described in ‘Materials and methods’. Data presented are averages of three experiments, each conducted in duplicates (±s.e.). ^**^P<0.01; ^***^P<0.001.

In order to strengthen the Western blot results, we further examined the activity of the above caspases using a colorimetric detection assay kit with specific caspase substrates, as described in ‘Materials and methods’. Figure 3

demonstrates the activity of both effector and initiator caspases in PC3 cells treated with DIM.

Our results indicate a significant increase in the activity of caspase 9 in these cells. The activity of this enzyme was twice that of the control when cells were exposed to DIM for 16 h, and it increased with time and reached a level five-fold higher than that of the control after 24 h, nine-fold higher after 48 h (*P*<0.01) and 16-fold higher after 72 h (*P*<0.01). The activity of caspase 8 also increased in these cells when exposed to DIM, starting from 24 h (twice that of the control), and reached a level eight times higher than that of the control after 72 h.

The results also show a significant increase in the activity of caspases 3 and 6, in a time-dependent manner. These caspase activities were found to be more than 5 (*P*<0.01), 15 (*P*<0.001) and 30 (*P*<0.001) times higher when the PC3 cells were exposed to DIM for 16–24, 48 and 72 h, respectively, in comparison to the controls.

### Release of cytochrome *c* to the cytosol

**Figure 4:**
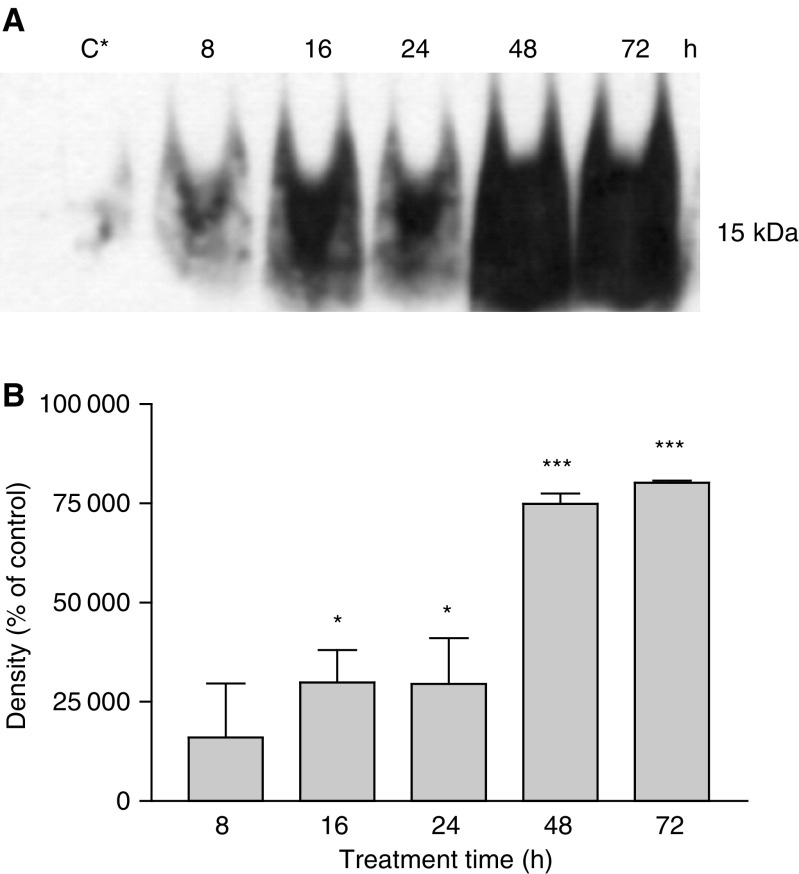
Release of cytochrome *c* to the cytosol in PC3 cells treated with 75 *μ*M DIM for 8–72 h. Cells were treated with DIM and their cytosol protein fraction was extracted, separated on SDS–PAGE and exposed to specific antibody using Western blotting, as described in ‘Materials and methods’. (**A**) Western blotting results. C^*^ denotes a representative control group. The figure shown is a representative of three independent experiments. (**B**) Densitometer results. Data presented are averages of three independent experiments (±s.e.) and are expressed as percentages of the respective controls. ^*^P<0.05; ^***^P<0.001.

Cytochrome *c* is a mitochondrial protein, which is released to the cytosol in response to a variety of apoptotic signals and promotes caspase cascade activation through caspase 9 (Nunez *et al*, 1998). Since our results indicated strong evidence for the activation of caspase 9 in PC3 cells after treatment with DIM, we examined whether the apoptotic response in these cells involves the translocation of cytochrome *c* from the mitochondria to the cytosol. For this purpose, the protein levels of cytochrome *c* in the cytosol were examined using Western blotting analysis and specific monoclonal antibody, as described in ‘Materials and methods’. The results indicated that treatment of the cells with 75 *μ*M DIM induced cytochrome *c* release to the cytosol after 8 h (Figure 4A

). This release rose with time of exposure, until reaching a maximum after 48 h. In the control group, only a minimal leakage of this protein was observed. Quantitative results, using a densitometeric analysis, displayed the significance of this protein release (Figure 4B). Treating cells with DIM for 16 and 24 h caused a significant release of cytochrome *c* to the cytosol (*P*<0.05) and this release was greatly increased with exposure times of 48 and 72 h (*P*<0.001).

**Figure 5 fig5:**
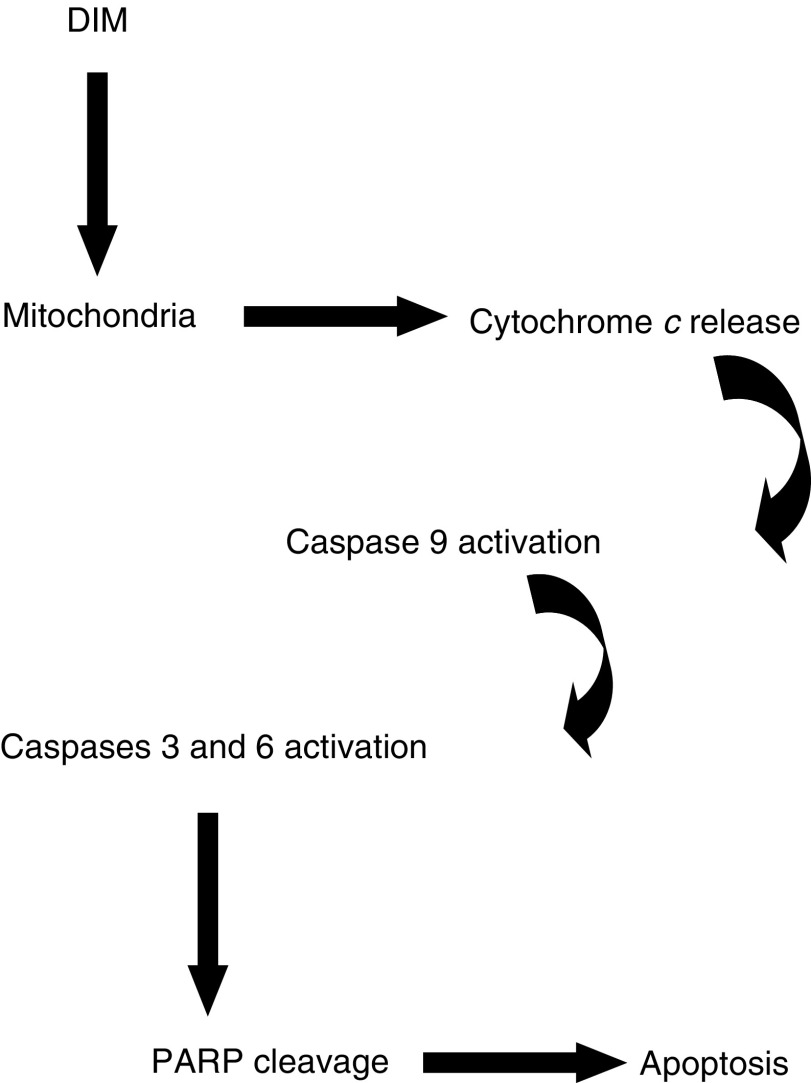
Schematic description of the apoptotic pathway in PC3 cells treated with DIM. Exposure of PC3 cells to DIM triggers the release of cytochrome *c* from the mitochondria. As a result, initiator caspase 9 is cleaved and activated, and cleaves and activates the effector caspases 3 and 6. These caspases cleave vital cell proteins such as PARP and cause the typical morphological and biochemical characteristics of apoptosis in these cells.

Actin level was used as a reference of a standard protein occurring naturally in these cells ([Fig fig5]

Figure 6

**Figure 6:**
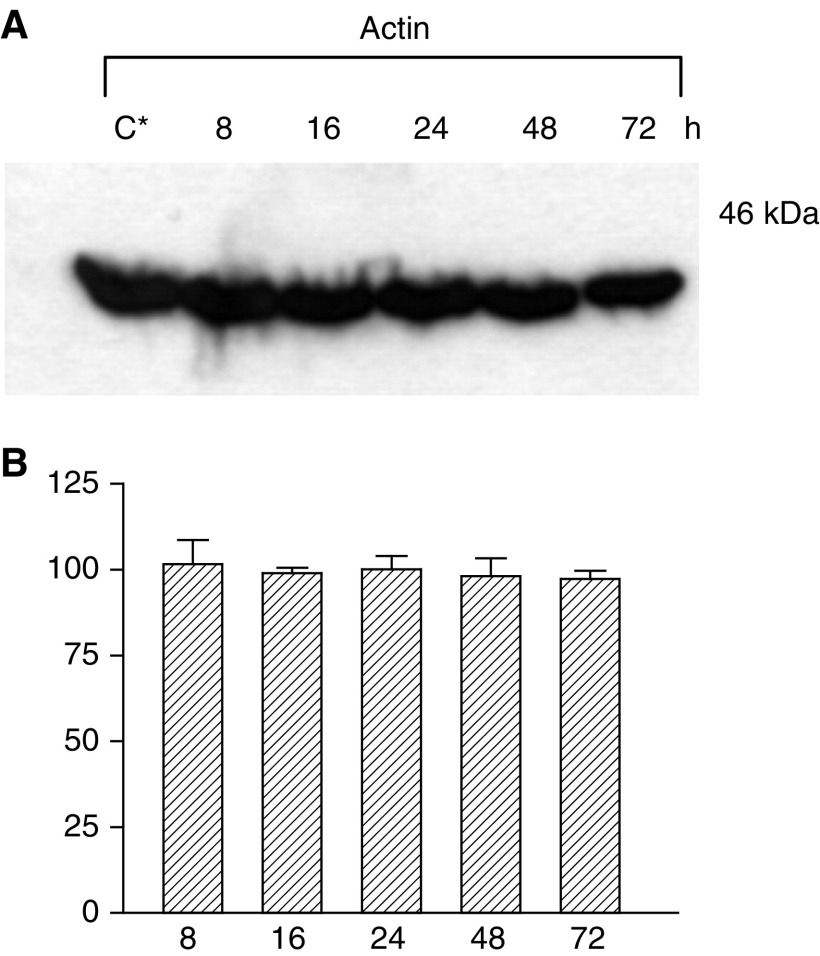
Actin levels following treatment of PC3 cells with 75 *μ*M DIM for 8–72 h. The experimental details and the designations of letters (**A**–**C**) were as described for Figure 1.

). The levels of this protein were equal in each of the samples.

## DISCUSSION

In the present study, we attempted to explore the mechanism of action involved in the beneficial effects of the indole derivative, DIM, on human androgen-independent prostate cancer cell line, PC3. We have previously reported that this indole derivative has a suppressive effect on the growth of prostate as well as breast cancer cell lines. This effect was mediated through the induction of an apoptotic process, as was observed through apoptotic morphological changes and the appearance of a typical DNA ladder within treated cells (Ge *et al*, 1996; Fares *et al*, 1998; Kedmi *et al*, 2003). Furthermore, we found that induction of apoptosis in PC3 cells by DIM occurred through a p53-, bax-, bcl-2- and fasL-independent pathway (Kedmi *et al*, 2003). In this study, we examined the levels and activities of several caspases in the same cells in response to DIM exposure. Caspases are cysteine proteases which are formed constitutively in the cells and are normally present as inactive proenzymes. Caspases are activated during apoptosis in a self-amplifying cascade (Saraste and Pulkki, 2000). Activation of upstream or initiator caspases, such as caspases 8, 9 and 10, by proapoptotic signals leads to the proteolytic activation of downstream or effector caspases 3, 6 and 7. The effector caspases cleave a set of vital proteins and, thus, initiate and execute the apoptotic degradation of the cell with the typical morphological and biochemical features. Two major pathways of caspase cascade activation have been characterised. One is initiated by ligation of death receptors and the activation of caspase 8. In the other, mitochondrial pathway, cytochrome *c* is released from mitochondria in response to a variety of apoptotic stimuli. In the cytosol, cytochrome *c* can bind to apaf-1 and, in the presence of dATP or ATP, it activates caspase 9 (Nunez *et al*, 1998; Saraste and Pulkki, 2000).

In the current study, we provide evidence that the induction of apoptosis occurring in PC3 cells by DIM is exerted through the mitochondrial pathway. Schematic diagram is presented in Figure 5. DIM triggers cytochrome *c* translocation from the mitochondria to the cytosol (Figure 4), which promotes the activation of caspase 9, that activates in turn caspases 3 and 6 (Figures 1, 2 and 3) in a time-dependent manner. These effector caspases are responsible for the cleaving of vital cell proteins such as the PARP protein (Kaufmann *et al*, 1993). We have previously shown the cleavage of this protein in these cells after exposure to DIM (Kedmi *et al*, 2003). In addition, there is a slight activation of caspase 8 in these cells as well, which may amplify the apoptotic process. It has previously been reported that caspase 8 may cleave the proapoptotic bcl-2 family member, bid, which translocates to the mitochondria, where it triggers cytochrome *c* release and the activation of apaf-1/caspase 9 pathway (Lou *et al*, 1998).

Several investigations have attempted to characterise the pathways involved in the apoptotic responses in several cancer cells exposed to indole derivatives, which are found in cruciferous vegetables. These studies found that such compounds have pleiotropic anticarcinogenic activities and may affect many biochemical pathways. Bonnesen *et al* (2001) demonstrated that indole compounds originating from crucifers prevent colon cancer cell lines growth through the induction of several detoxification enzymes. Leong *et al* (2001) reported cytostatic effects of DIM in human endometrial cancer cells, which was mediated by the induction of a transforming growth factor-*α* expression and signal transduction pathway. Carter *et al* (2002) showed, using cDNA microarrays, that DIM altered the expression of more than 100 genes by at least two-fold in human cervical cancer cells. Many of the stimulated genes encode to transcription factors and proteins involved in signalling, stress response and growth. Recently, Li *et al* (2003) reported the gene expression profiles of I3C- and DIM-treated PC3 cells, as was determined by cDNA microarray analysis. These researchers found that these indole derivatives up- and downregulate the expression of a large number of genes, which are significant in the regulation of critical events such as cell growth, cell cycle and apoptosis. Chinni and Sarkar (2002) revealed that I3C-induced apoptosis in PC3 cells is partly mediated by the inhibition of Akt activation pathway and downregulation of BAD and bcl-x_L_. However, it has been reported that I3C is unstable in tissue culture medium and under acidic conditions, and is partially converted to the very stable compound, DIM (Bradfield and Bjeldanes, 1987; Ge *et al*, 1999; Staub *et al*, 2002). Therefore, the *in vitro* effect of I3C may be partially attributed to its dimeric product, DIM. Hence, Chinni and Sarkar's (2002) study on Akt inactivation may support our findings regarding the apoptotic pathway of DIM in PC3 cell lines. Akt, a protein kinase, was shown to inhibit apoptosis and the processing of pro-caspases to their active forms, by delaying mitochondrial changes. Akt promotes cell survival by inhibiting the release of cytochrome *c* from the mitochondria (Kennedy *et al*, 1999) and phosphorylating and inactivating caspase 9 (Cardone *et al*, 1998).

In conclusion, in this study we provide evidence that the indole derivative, DIM, induces apoptosis in human PC3 prostate cancer cells, through the mitochondrial pathway. Our results may contribute to a better understanding of the molecular mechanisms by which DIM exerts its effects in prostate cancer cells and tumours. These findings may lead to the development of new therapeutic strategies for the treatment of androgen-independent prostate malignancy, for which there is no effective life-prolonging therapy.
